# Cross-cultural adaptation and multicentric validation of the Italian version of the Simplified Evaluation of CONsciousness Disorders (SECONDs)

**DOI:** 10.1371/journal.pone.0317626

**Published:** 2025-02-10

**Authors:** Bahia Hakiki, Silvia Pancani, Agnese De Nisco, Anna Maria Romoli, Francesca Draghi, Daniela Maccanti, Anna Estraneo, Alfonso Magliacano, Marcella Spinola, Cinzia Fasano, Matilde Leonardi, Martina Cacciatore, Francesca Giulia Magnani, Davide Sattin, Camilla Ippoliti, Filippo Barbadoro, Antonello Grippo, Claudio Macchi, Charlotte Martial, Olivia Gosseries, Francesca Cecchi

**Affiliations:** 1 Istituto di Ricovero e Cura a Carattere Scientifico (IRCCS) Fondazione Don Carlo Gnocchi, Firenze, Italy; 2 Department of Experimental and Clinical Medicine, University of Florence, Florence, Italy; 3 Polo Specialistico Riabilitativo, Fondazione Don Carlo Gnocchi ONLUS, Sant’Angelo dei Lombardi, AV, Italy; 4 SC Neurologia, Salute Pubblica, Disabilità—Fondazione IRCCS Istituto Neurologico Carlo Besta, Milan, Italy; 5 Istituti Clinici Scientifici Maugeri IRCCS, Milan, Italy; 6 Coma Science Group, GIGA-Consciousness, University of Liège, Liège, Belgium; 7 Centre du Cerveau, University Hospital of Liège, Liège, Belgium; CHU Nantes, FRANCE

## Abstract

**Introduction:**

The Coma Recovery Scale-Revised (CRS-R) is the recommended tool to assess consciousness in patients with prolonged Disorders of Consciousness (pDoC). However, the time needed to administer it may limit its use. A shorter tool has been validated: the Simplified Evaluation of CONsciousness Disorders (SECONDs). This multicentre study aimed to develop and validate a cross-cultural adaptation of the SECONDs into Italian.

**Methods:**

An interdisciplinary expert team, from both Fondazione Don Carlo Gnocchi and Istituto Neurologico Carlo Besta, led the translation processes. Independent certified translators were also involved in a blinded modality. Patients diagnosed with Unresponsive Wakefulness Syndrome (UWS) or Minimally Conscious State (MCS) admitted to 3 Italian rehabilitation units were enrolled. The CRS-R and SECONDs were administered in 5 sessions over two weeks by 3 blinded examiners at each center (3 times, with 2 sessions conducted by the same examiner). Weighted Fleiss’ kappa and Spearman correlation coefficients were used to assess intrarater and interrater reliability and concurrent validity.

**Results:**

Sixty adults with pDoC were assessed: 23 women; median age: 64 years; 14 trauma, median post-onset time: 2 months. Intrarater and interrater reliability showed almost perfect agreement (kappa coefficients 0.968 and 0.935, respectively; p<0.001). The comparison of CRS-R vs. SECONDs on the same day or the best out of 5 SECONDs/CRS-R led to a substantial to almost perfect agreement both for the total score of the CRS-R and the SECONDs’ Additional Index (ρ = 0.772–1.000; p<0.001) and for the consciousness diagnosis (k = 0.784–0.935; p<0.001). The disagreement rate between the overall best diagnosis of the SECONDs and the best CRS-R diagnosis was 6.7%.

**Conclusion:**

The Italian version of the SECONDs has been cross-culturally adapted to serve as a shorter assessment tool for the diagnosis of pDoC. Our study shows its excellent reliability and concurrent validity when compared to the CRS-R.

## Introduction

Severe acquired brain injury of any aetiology may lead to disorders of consciousness (DoC), which are currently mainly classified based on behavioural responses into the Unresponsive Wakefulness Syndrome (UWS: awake but unaware patients) and the Minimally Conscious State (MCS: patients with minimal but reproducible behavioural signs of consciousness). MCS is further divided into MCS- and MCS+ based on the absence or presence of language comprehension [1]. When the DoC persists for more than 28 days, it is defined as a prolonged DoC (pDoC). When patients recover functional communication or functional use of objects, they have emerged from the MCS (EMCS) [2]. The misdiagnosis rate has been shown to be high (up to 40%) when validated tools are not used for multiple assessments [3]. Therefore, since the diagnosis can influence the patient’s care pathway, as to treatment choices, end-of-life decisions, definition of the rehabilitation path, and information given to relatives, the use of standardized, sensitive, and validated scales is fundamental [4, 5]. The Coma Recovery Scale-Revised (CRS-R) [6, 7] is currently the recommended diagnostic scale [8], however, it is time consuming, requires expertise to be administered, and provides a total score that cannot be associated with a single diagnosis (e.g., both UWS and MCS can have a total score of 7) [9, 10]. To reduce the misdiagnosis risk due to clinical variability, recent recommendations highlight the importance of repeating CRS-R evaluations and involving caregivers during its administration, whether feasible [11]. When these recommendations are applied, the CRS-R administration becomes even more time consuming, with limited feasibility in the current clinical practice [5, 11].

The Simplified Evaluation of CONsciousness Disorders (SECONDs) was elaborated by Aubinet and colleagues [12] on the basis of the CRS-R. It was inspired by the most frequently observed signs of consciousness when using the CRS-R for the diagnosis of pDoC. The SECONDs includes 8 items (6 mandatory and 2 conditional) of increasing complexity total score ranges from 0 to 8 and each score is associated with a diagnosis: Coma (0), UWS (1), MCS- (2–5), MCS+ (6–7), EMCS (8). Importantly, the SECONDs also provides an additional index, developed to give a more accurate account of the behaviours observed during the assessment, allowing the monitoring of a patient’s level of consciousness over time [13]. An additional-index point obtained for each conditional item can be added to calculate the additional index score, ranging from 0 to 100. Zero additional-index point should be scored for non-administered conditional items (e.g., pain localization when command-following is present) or unsuccessful items (i.e., when the criteria to score the item are not met). The SECONDs administration required a median time of 7 minutes vs. 21 minutes required by the CRS-R in the same study, and the tool was reported as easy-to-use [13]. Thus, its repetition over time appeared more feasible compared to the CRS-R. Being easy to handle, the SECONDs has been proposed to facilitate the consciousness assessment in patients with DoC, thus reducing the risk of misdiagnosis in all clinical settings. Indeed, the SECONDs aims at fostering accurate assessment of patients with pDoC in all settings (from the intensive care units to outpatients’ consultation for patients with pDoC), and to promote the standardization of clinical research in this field from the acute to the chronic phase. The SECONDs has already been validated in French [12] and Mandarin [14], showing excellent psychometric properties.

This multicentric observational study aimed at developing a cross-cultural adaptation of the SECONDs into Italian and assessing its intrarater and interrater reliability and concurrent validity.

## Material and methods

### Translation and back-translation (Phase 1)

**Fig 1:**
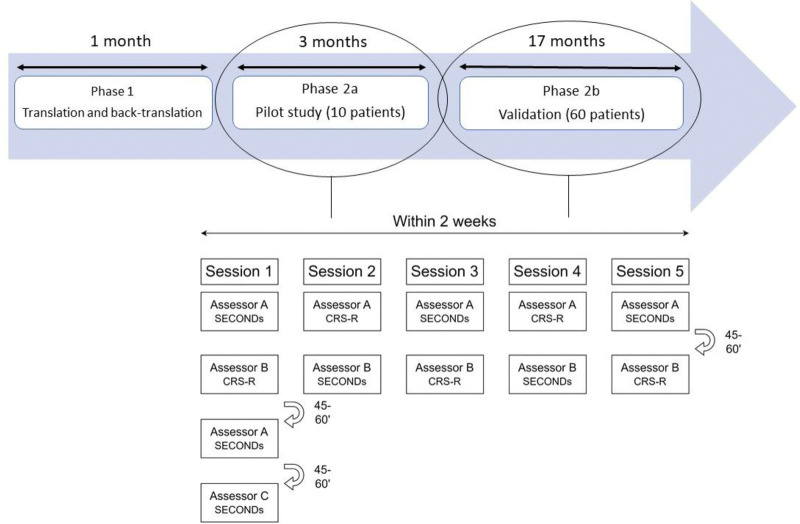
SECONDs validation process. Procedure for the validation of the Simplified Evaluation of CONsciousness Disorders (SECONDs). For each participant, a SECONDs was performed either before or after a Coma Recovery Scale-Revised (CRS-R) assessment and this order was randomised in 5 sessions. Five one-day sessions were performed over 2 weeks. There were 3 assessors (A, B, and C) who evaluated the patients.

The first phase of the study consisted of the cross-cultural adaptation of the SECONDs into Italian (Phase 1). The cross-cultural adaptation, referring to the process of considering any differences between the source and the target culture so as to maintain equivalence in meaning [15], encompasses the process that looks at both translation and cultural adaptation issues [16]. Indeed, it is now recognized that if measures are to be used across cultures, the items must not only be translated linguistically well, but also must be adapted culturally to maintain the content validity of the instrument at a conceptual level across different cultures [16]. In this study, the cross-cultural adaptation was carried out according to internationally accepted procedures [16] by providing a protocol of forward and backward translations and cultural adaptation. An interdisciplinary team with experience in rehabilitation and research on pDoC composed of 8 researchers including neurologists (BH, AE, AG, ML), physiatrist (FC) and neuropsychologists (FM, MC, DS), belonging to both the Fondazione Don Carlo Gnocchi and the Istituto Neurologico Carlo Besta led the translation processes. Independent certified translators were also involved in these two steps in a blinded modality. All the Italian researchers were fluent in both Italian and French languages. In addition, during this phase, we referred to a researcher (CM) from the Coma Science Group at the University of Liège (Belgium) who validated the original scale. Three Italian versions were produced (two by the expert team FC, AG and AE DS respectively and the other by the certified translators) from which a single Italian version was derived. To achieve a good equivalence of the translation [17], the Italian version was discussed and cross-culturally adapted by 3 members of the expert team not involved in the forward translation (AE, DS, FM). The adapted version was then back translated into French by another certified professional translator and by two members of the expert team (BH, MC), blind to the original French version. Again, the French version was discussed by members of the team experts and compared with the original version (BH, MC, ML). Finally, all discrepancies were discussed among all the experts involved in the translation with the aim of determining the conceptual, semantic, and operational equivalence of the translated scale (Fig 1) to produce the final Italian version of the SECONDs.

### The validation (Phase 2)

From November 2021 to October 2023, patients with a pDoC occurred within three months from the acute event were prospectively recruited at the IRCCS Fondazione Don Gnocchi of Florence and Fondazione Don Gnocchi of Sant’Angelo dei Lombardi, whereas chronic patients with pDoC were recruited at the IRCCS Fondazione Carlo Besta of Milan during outpatients’ evaluations (median time from event 7 [IQR 12] months). Inclusion criteria were the presence of severe acquired brain injury leading to a pDoC, ≥18 years old, no history of previous neurological or psychiatric deficits, Italian as mother tongue, and medical stability (e.g., absence of mechanical ventilation, sedation, infection).

This study was approved by the ethics committee of each unit: IRCCS Fondazione Don Gnocchi Florence: 20239_oss; Fondazione Don Gnocchi of Sant’Angelo dei Lombardi: 1918 (28/09/2022); IRCCS Fondazione Carlo Besta: 01/2022, and registered on clinicaltrial.gov (NCT05714215). Written informed consent was obtained from the participants’ legal surrogates. The study followed the principles of the Declaration of Helsinki.

Overall, a team of 11 examiners (Florence: 1 neurologist, 2 speech therapists, and 1 physical therapist; Sant’Angelo: 1 neuropsychologist and 2 speech therapists; Besta: 4 neuropsychologists) performed all the assessments. All the examiners were experts in the administration of the CRS-R scale and they received instructions in the administration of the SECONDs through a group training session, carried out via videoconference performed by the promoter (Fondazione Don Gnocchi Florence) with the support of the Coma Science Group members involved in this study [13] and lasting 45 minutes.

According to the procedure established by the authors of the original scale [12] and already used in the validation of the Mandarin version of the SECONDs [14], for each patient, assessments were completed within 2 weeks: 5 CRS-R were administered to obtain a reliable diagnosis [18] as well as 7 SECONDs, all performed by 3 different assessors (A, B, C). A total of 5 sessions were performed. Session 1 included one CRS-R performed by only one assessor (assessor B; to measure concurrent validity) and three SECONDs evaluations performed by both assessors A and C (to measure intrarater reliability and inter-rater reliability), providing a time break of 45–60 minutes between each assessment to avoid fatigue/arousal fluctuations and minimise the recall bias in the assessment of intrarater reliability. The remaining four sessions, from 2 to 5, included each one SECONDs evaluation and one CRS-R evaluation (to measure concurrent validity) and were performed by assessors A and B. Each SECONDs was performed alternatively either before or after the CRS-R assessment, again with a time break of 45–60 minutes between assessments. For the duration of the protocol, all three assessors were blind to all the scores and the consciousness diagnosis of the other assessors (Fig 1).

The translation validity has been verified using a pilot validation/pre-test (Phase 2A). To identify scale items presenting difficulties in administration reported either from examiners or patients, due to linguistic inaccuracies in the translated version between raters, a non-definitive translated version of the SECONDs was administered to the first 10 recruited patients. Finally, the validation was completed on the whole sample of the study, consisting of 60 patients (Phase 2B).

### Statistical analysis

#### Sample size

For the pilot validation study (Phase 2A), the enrolment of 10 patients was planned. This number was established based on the indications provided in the literature for transcultural validation procedures [19, 20], which suggest the use of at least 10–15 participants to evaluate whether the translated scale presents questions that are difficult to answer, is ambiguous, difficult to understand or presents inadequate language.

The sample size for Phase 2B was calculated based on the correlation between the SECONDs and the CRS-R total score, evaluated using the Spearman’s rank correlation coefficient (ρ). For this analysis, according to the literature, the correlation was assessed as very strong in the presence of ρ 0.9–1.0, strong if ρ 0.7–0.89, moderate in the presence of ρ 0.4–0.69, and weak in the presence of ρ <0.4 [21]. The calculation of the sample was done using the G*Power software (version 3.1.9.6), assuming as a null hypothesis (H0) the absence of correlation (ρ = 0) and as an alternative hypothesis (H1) the existence of a correlation between the two scales (ρ ≠ 0). The sample was estimated using a two-way test and predicting a correlation between the scales of at least 0.5 (based on the correlation observed between the original version of the SECONDs and the CRS-R [12]), a type I error of 0.01, and a power of 90%.

The required sample was equal to 50 subjects, to which 20% was added to deal with any dropouts, reaching a total of 60 subjects. This estimate aligns with the number of subjects included in the validation study of the original version of the SECONDs in which 57 patients were enrolled [12].

Descriptive data were reported through mean and standard deviation (or median and interquartile range, depending on the normality of the data distribution) for quantitative variables, and as frequencies and percentages for dichotomous and categorical variables. Median values and IQR were reported when data did not meet the normality assumption.

The linear weighted Fleiss’ kappa coefficient and percentages of observed agreement (OA) were used to estimate the concurrent validity between the diagnosis obtained using the CRS-R and the SECONDs on the same day and the best CRS-R and the best SECONDs diagnosis obtained over the five evaluations. The same coefficients were used to estimate the intrarater reliability (by comparing the diagnosis obtained by the same examiner on different observations) and interrater reliability (by comparing the diagnosis obtained by different examiners on the same day). The agreement was considered poor if k < 0, slight if 0<k<0.2, fair if 0.21<k<0.4, moderate if 0.41<k<0.6, substantial if 0.61<k<0.8, and almost perfect if 0.81<k<1 [22]. The analyses were performed both considering MCS as a unique class and by subclassifying the MCS patients into MCS+ and MCS-.

The Spearman’s rank correlation coefficient was calculated between the best SECONDs and the best CRS-R scores obtained over the five evaluations as well as the scores of the SECONDs and the CRS-R recorded on the same day. For the SECONDs total score and the SECONDs additional index, the one-way Intraclass Correlation Coefficient for single measures (ICC1,1) was computed for inter and intrarater reliability. Statistical analyses were conducted using SPSS (Version 28.0. Armonk, NY: IBM Corp). Results were considered significant at p < 0.05.

## Results

### Translation and back-translation (Phase 1)

A term, “item conditionnel”, used to describe two items (pain and communication) was subjected to a cross-cultural adaptation: in the instructions, the French version referred to a”conditional item” to indicate that the item should be administered only under certain specific conditions. As in Italian “item condizionale” was found to be unclear by the expert team because (“condizionale” in Italian is the name of the verbal mode “conditional”), for a clearer translation of this item, it has been opted for “item sottomesso a condizione” that is item subject to a condition.

### Validation (Phase 2)

The standardised multi-step translation process was carried out, and the cross-culturally adapted Italian version of SECONDs was administered to 10 patients (Phase 2A). All members were asked to report any difficulties in administering the scale during this phase of the study to the promotor (IRCCS-Fondazione Don Gnocchi Florence). Since no difficulties were reported and no need for item reformulation occurred, this version of SECONDs was adopted as final, including instructions and additional index, as reported in the Appendix, and used for the validation study.

Overall, sixty patients were enrolled, and completed the study, no patients dropped out of the study: the median [IQR] age was 64 [IQR = 34] years old, and 23 (38.3%) patients were women. One-third of the participants had a haemorrhagic brain injury (20 patients, 33.3%). Overall, the median time between the brain injury and the first evaluation was 2 months [IQR = 6]; specifically, the post-acute subgroup showed a median time post-onset of 2 months [IQR = 1], whilst the chronic subgroup was characterised by a median time post-onset of 9 [IQR = 9] months. All participants data and descriptive statistics are shown in Table 1.

**Table 1:** Clinical and demographic characteristics of the study sample.

	Total sample (N = 60)	Post-acute (N = 37)	Chronic (N = 23)	p-value
	N (%)	N (%)	N (%)	
	median [IQR]	median [IQR]	median [IQR]	
Age (years)	64 [34]	68 [38]	53 [29]	0.079
TPO (months)	2 [6]	2 [1]	9 [9]	<0.001
Sex (F)	23 (38.3%)	16 (43.2%)	7 (30.4%)	0.321
Aetiology				0.093
Traumatic	14 (23.3%)	9 (24.3%)	5 (21.7%)	
Anoxic	11 (18.3%)	3 (8.1%)	8 (34.8%)	
Ischemic	8 (13.3%)	6 (16.2%)	2 (8.7%)	
Haemorrhagic	20 (33.3%)	13 (35.1%)	7 (30.4%)	
Other	7 (11.7%)	6 (16.2%)	1 (4.3%)	

TPO: time Post-Onset; IQR: interquartile range

### Intrarater reliability and correlation

When considering the MCS as a single category, the weighted kappa of two SECONDs assessments by the same assessor was 0.951 (p<0.001). When considering MCS- and MCS+ as separate categories, the results of Assessor A’s first assessment in session 1 were: 3 Coma; 33 UWS; 10 MCS-; 11 MCS+; and 3 EMCS. The results of Assessor A’s second assessment in session 1 were: 3 Coma; 31 UWS; 12 MCS-; 11 MCS+; and 3 EMCS (Table 2). The weighted kappa of two times SECONDs assessments by this same assessor was 0.968 (p<0.001). This demonstrates an almost perfect [18] intrarater reliability when the same evaluator rated the same participant at different times of the same day. The observed agreement was 0.97. The intrarater ICC was 0.972 (p<0.001) according to the SECONDs total score and ICC = 0.976 (p<0.001) according to the SECONDs additional index score.

**Table 2:** Intrarater reliability and correlation, OA: Observed agreement.

k = 0.968, p<0.001; OA = 0.97		SECONDs (Assessor A -1^st^ day, 2^nd^ assessment)							
		Coma	UWS		MCS-		MCS+		EMCS
SECONDs (Assessor A– 1^st^ day, 1^st^ assessment)	Coma	3	0		0		0		0
	UWS	0	31		2		0		0
	MCS-	0	0		10		0		0
	MCS+	0	0		0		11		0
	EMCS	0	0		0		0		3
k = 0.951, p<0.001; OA = 0.97		SECONDs (Assessor A -1^st^ day, 2^nd^ assessment)							
		Coma		UWS		MCS		EMCS	
SECONDs (Assessor A– 1^st^ day, 1^st^ assessment)	Coma	3		0		0		0	
	UWS	0		31		2		0	
	MCS	0		0		21		0	
	EMCS	0		0		0		3	

### Interrater reliability and correlation

Interrater reliability for the SECONDs was almost perfect [22] (k = 0.936, p<0.001; OA = 0.93) either considering MCS as a single category or MCS- and MCS+ separately (k = 0.928, p<0.001; OA = 0.95). This indicates that the SECONDs could reliably assess the same participant when performed by different assessors (Table 3). The interrater ICC was 0.962 (p<0.001) according to the SECONDs total score and ICC = 0.962 (p<0.001) according to the SECONDs additional index score.

**Table 3:** Interrater reliability and correlation, OA: Observed agreement.

k = 0.936, p<0.001; OA = 0.93		SECONDs (Assessor C)							
		Coma	UWS		MCS-		MCS+		EMCS
SECONDs (Assessor A– 1^st^ day, 1^st^ assessment)	Coma	3	0		0		0		0
	UWS	1	30		2		0		0
	MCS-	0	0		10		0		0
	MCS+	0	0		1		10		0
	EMCS	0	0		0		0		3
k = 0.928, p<0.001; OA = 0.95		SECONDs (Assessor C)							
		Coma		UWS		MCS		EMCS	
SECONDs (Assessor A– 1^st^ day, 1^st^ assessment)	Coma	3		0		0		0	
	UWS	1		30		2		0	
	MCS	0		0		21		0	
	EMCS	0		0		0		3	

### Concurrent validity between CRS-R and SECONDs

The concurrent validity between diagnoses made with the CRS-R and the SECONDs on the same day over the five iterations was substantial to almost perfect, both considering MCS as a single category (k range: 0.772–1.000; p<0.001; OA range: 0.90–1.00) and considering MCS- and MCS+ as distinct categories (k range: 0.784–0.935; p<0.001; OA range: 0.87–0.95; Results from 1^st^ day shown in Table 4). The concurrent validity between the best CRS-R and the best SECONDs diagnosis (from all 5 iterations; Table 5) was also almost perfect for a single MCS category (k = 0.948, p<0.001; OA = 0.97) and when considering MCS- and MCS+ sub-categories (k = 0.919, p<0.001; OA = 0.93; Table 4A).

**Table 4:** Concurrent validity between CRS-R and SECONDs diagnoses.

A	Same-day SECONDs						Best SECONDs				
	UWS	MCS-		MCS+	EMCS		UWS	MCS-		MCS+	EMCS
CRS-R 1st day						CRS-R best					
UWS	35	0		1	0	UWS	32	0		1	0
MCS-	0	10		1	0	MCS-	0	10		0	0
MCS+	1	0		8	0	MCS+	0	2		10	0
EMCS	0	0		1	3	EMCS	0	0		1	4
B			Same-day SECONDs Additional Index						Best SECONDs Additional Index		
CRS-R 1st day			ρ = 0.839 p-value <0.001			CRS-R Best			ρ = 0.895 p-value <0.001		

Comparison between diagnoses made with the CRS-R and the SECONDs on the same day and between A: the best CRS-R and the best SECONDs diagnosis (from all 5 iterations); B: the best CRS-R score and the best SECONDs additional index from all 5 iterations.

**Table 5:** Demographic and diagnosis data for the included patients.

Participant ID	Age	Sex	DoC Aetiology	Time between injury and first session (months)	Best CRS-R diagnosis	Best SECONDs diagnosis
1	75	F	Haemorrhagic	3	MCS-	MCS-
2	39	M	Traumatic	1	UWS	UWS
3	36	F	Anoxic	3	UWS	UWS
4	71	M	Anoxic	19	UWS	UWS
5	38	F	Anoxic	27	UWS	UWS
6	72	F	Ischemic	1	UWS	UWS
7	74	M	Ischemic	2	MCS+	MCS+
8	29	M	Traumatic	2	UWS	UWS
9	43	M	Anoxic	2	UWS	UWS
10	19	F	Traumatic	3	UWS	UWS
11	28	M	Traumatic	2	UWS	UWS
12	53	M	Other	13	UWS	UWS
13	48	F	Haemorrhagic	9	MCS-	MCS-
14	61	M	Anoxic	59	UWS	UWS
15	69	F	Haemorrhagic	2	UWS	UWS
16	23	F	Traumatic	3	UWS	UWS
17	73	F	Other	1	UWS	UWS
18	64	F	Other	1	UWS	UWS
19	78	F	Traumatic	1	UWS	UWS
20	49	F	Other	2	MCS-	MCS-
21	34	M	Other	26 days	EMCS	EMCS
22	65	M	Haemorrhagic	2	MCS-	MCS-
23	80	M	Haemorrhagic	2	**MCS+**	**MCS-**
24	29	M	Anoxic	13	MCS+	MCS+
25	83	F	Haemorrhagic	1	UWS	UWS
26	44	F	Other	2	EMCS	EMCS
27	86	M	Traumatic	2	MCS-	MCS-
28	86	M	Traumatic	1	**MCS+**	**MCS-**
29	76	M	Haemorrhagic	4	UWS	UWS
30	67	F	Ischemic	1	UWS	UWS
31		M	Haemorrhagic	27 days	MCS+	MCS+
32	69	M	Anoxic	6	MCS-	MCS-
33	34	M	Traumatic	6	UWS	UWS
34	58	M	Anoxic	7	UWS	UWS
35	65	F	Haemorrhagic	6	EMCS	EMCS
36	21	M	Traumatic	7	MCS+	MCS+
37	84	F	Haemorrhagic	2	MCS+	MCS+
38	48	F	Other	28 days	MCS+	MCS+
39	82	M	Haemorrhagic	2	UWS	UWS
40	52	M	Haemorrhagic	19	MCS+	MCS+
41	37	M	Traumatic	3	UWS	UWS
42	48	F	Haemorrhagic	32	EMCS	EMCS
43	72	M	Anoxic	2	UWS	UWS
44	76	F	Ischemic	4	UWS	UWS
45	78	M	Haemorrhagic	1	UWS	UWS
46	36	M	Ischemic	1	UWS	UWS
47	80	M	Haemorrhagic	2	MCS-	MCS-
48	65	F	Anoxic	8	UWS	UWS
49	67	M	Anoxic	6	MCS+	MCS+
50	73	M	Haemorrhagic	2	UWS	UWS
51	67	M	Ischemic	2	UWS	UWS
52	70	M	Ischemic	1	MCS-	MCS-
53	50	M	Haemorrhagic	4	**UWS**	**MCS+**
54	79	M	Haemorrhagic	1	UWS	UWS
55	55	M	Traumatic	15	MCS+	MCS+
56	52	M	Ischemic	15	MCS-	MCS-
57	32	M	Traumatic	11	MCS+	MCS+
58	70	F	Haemorrhagic	12	**EMCS**	**MCS+**
59	34	M	Traumatic	9	UWS	UWS
60	53	F	Haemorrhagic	2	MCS-	MCS-

When there was a discrepancy between the best diagnosis made by the CRS-R vs. SECONDs, it is illustrated in bold.

The correlation between the CRS-R score and the SECONDs additional index recorded on the same day and between the best of the five iterations was strong (ρ = 0.839, p<0.001 and ρ = 0.895, p<0.001; Table 4B).

### Concurrent validity between CRS-R and SECONDs including the additional index

Concurrent validity between the CRS-R total score and the SECONDs additional index total score was also investigated. The correlation with the CRS-R on the same day ranged from 0.716 to 0.847 for the SECONDs total score and from 0.723 to 0.839 for the additional index. Similarly, a substantial concurrent validity was observed between the CRS-R and SECONDs best total score recorded over the five evaluations (ρ = 0.880, p<0.001) and between the CRS-R and the SECONDs best additional index recorded over the five evaluations (ρ = 0.895, p<0.001).

## Discussion

The SECONDs was developed to provide a short but reliable tool to accurately and quickly assess the state of consciousness in patients with DoC in daily care. The development of this scale was based on the CRS-R [23, 24], the currently recommended tool for the clinical assessment of consciousness in patients with DoC [8]. To adopt this scale in the Italian context, a multicentric validation of the Italian version of the SECONDs was developed and provided. The present findings showed an almost perfect concurrent validity between the SECONDs and CRS-R diagnoses in individuals with pDoC, as well as almost perfect agreement in terms of intrarater and interrater reliability, as observed in the original validation of the SECONDs [12].

Differently from previous validations of the SECONDs [12, 14] though, in the present work, the additional index was included in the analyses to compare the CRS-R and the SECONDs. Substantial concurrent validity between CRS-R and SECONDs was observed both considering the diagnostic categories and the total scores (CRS-R total score vs SECONDs’ additional index) in individuals with pDoC. Also, the concurrent validity between CRS-R and SECONDs remained substantial both merging patients with MCS- and MCS+ in a unique category and considering them separately. The disagreement rate between the overall best diagnosis of the SECONDs and the best CRS-R diagnosis was 6.7%. This percentage is lower in comparison with the French validation study of the SECONDs (19%) [12] and the Mandarin one (29.8%) [14]. This difference could be partially explained by the inclusion of skilled assessors with at least 5 years of experience in the evaluation of pDoC patients.

By considering the level of consciousness detected by the CRS-R and the (best) SECONDs during the same day, a consistency was observed for 35 UWS, 10 MCS-, 8 MCS +, and 3 EMCS patients. Discrepancies emerged for 2 patients resulting in a MCS+ according to SECONDs but who were classified as UWS and MCS- by the CRS-R. On the other hand, one patient diagnosed in a MCS+ according to the CRS-R assessment resulted in an UWS at the SECONDs, and one patient who was diagnosed in an EMCS according to the CRS-R was in a MCS+ when considering the SECONDs. Analysing the discrepancies in the diagnosis derived from the CRS-R and SECONDs observed during the same day in these four patients, it was observed that they may be related to the intrinsic limitations of each scale. Patient n°31 resulted as MCS+ at the SECONDs since he was able to present response to command (shake the hand twice out of three commands and move a limb twice out of three commands). In the CRS-R assessment, the same patient was diagnosed in a MCS- (CRS-R total score = 12 (A2, V3, M5, O0, C0, Arou2)) since the scale requires at least three responses for scoring. Patient n°49 was diagnosed in a MCS+ with a CRS-R total score of 9 (A2, V1, M2, O3, C0, Arou1). At the SECONDs, his consciousness was classified as UWS in the three assessments during the first day since he was not able to show any response to command (item B), thus his capability to speak was probably not evaluated at all during the SECONDs assessment (the verbal function is possible but not specifically addressed in item G ‘Oriented behaviours’ of the SECONDs, it is required only for the assessment of conditional item C ‘Communication’). Patient n°53 resulted in UWS with a CRS-R total score of 5 (A0, V0, M2, O1, C0, Arou2). At the SECONDs, his consciousness was classified as MCS+ since he was able to avoid the painful stimulation which is not required during the CRS-R evaluation. Patient n°58 resulted in an EMCS at the CRS-R (total score 9: A3, V4, M6, O1, C1, Arou2) thanks to his functional object use. This ability was not tested by the SECONDs so that the consciousness level assigned to the patient was MCS+ (Intentional communication). The latter reason for discrepancy was also identified in the original SECONDs validation study [12].

Concerning the intrarater and interrater agreement instead, a discrepancy was observed in 2 patients resulting in UWS during the first assessment, and MCS- during the second one (Table 4), confirming that repetitive assessments, facilitated by this less time-consuming scale, improved the probability of detecting higher states of consciousness [5].

The SECONDs represents an easy-to-use scale to assess the consciousness state that, compared to the CRS-R scale, requires fewer materials (only a mirror), less time, and a shorter training (also available online). Its validation was carried out according to the European recommendations for the administration of the CRS-R such as the repeated administration of the scale and the use of a mirror for the visual fixation and pursuit. Also, the SECONDs allows to discriminate between MCS+ and MCS-. Other simplified assessment tools derived from the CRS-R were proposed to facilitate the consciousness’ stratification and save time for clinicians. The simplified CRS-R [25] included the 8 most commonly observed behaviours from the CRS-R scale. This scale was tested on 150 patients with anoxic, traumatic, vascular and tumoral DoC. Its administration’s duration was not specified in the study. Despite not testing the test-retest reliability, a good agreement level between the diagnostic outcomes of the CRS-R and simplified CRS-R was found. However, this scale was not able to distinguish between MCS+ and MCS- which is an important limitation for the prognosis formulation. Another tool to quickly assess consciousness in patients with DoC is the CRS-R For Accelerated Standardized Testing (CRSR- FAST) [26]. This scale has been tested in 45 traumatic patients hospitalised in intensive care units. It required about 6.5 minutes to be completed and showed good psychometric parameters. Yet, the validation protocol included a single administration of the CRS-R in contrast with what is strongly recommended by the European Academy of Neurology guideline on the diagnosis of coma and other DoC [5].

The SECONDs is potentially extendable to professionals working in other settings such as intensive care units. Shortening the assessment time is an invaluable advantage that allows clinicians to repeat the evaluation several times a day, thus reducing the misdiagnosis risk due to the arousal fluctuations in pDoC patients. The simplicity of SECONDs and the time gain could also promote collaboration between clinicians and family members, leading to more assessment sessions conducted jointly by both parties. This perspective presents different advantages. First, it would allow the use of highly emotionally loaded stimuli as relative’s voices are [27]. Second, it would reduce the discrepancy in the perception of consciousness between families and professionals [28], that often results in great suffering for patients’ relatives and places the clinician in stressful situations raising their burn-out risk [29]. Nevertheless, clinical assessment of consciousness is subject to a non-negligible risk of error, which is why current recommendations stressed the importance of combining clinical and instrumental evaluations [5]. This risk increases in certain clinical conditions [30] or when a covert consciousness [31] is suspected, e.g. due to the lesion site. Under the above-mentioned conditions, it would be desirable to prefer the CRS-R to the SECONDs in accordance with current recommendations [5].

While not replacing the reference scale for the diagnosis of consciousness in pDoC, which remains the CRS-R, the SECONDs could be integrated into the patient’s clinical assessments and would guide clinicians and patients’ family in optimizing therapeutic decisions in this challenging population, while also acting as a bridge between health professionals and family members during patient care.

This Italian validation of the SECONDs presents some limitations. The study was performed in rehabilitation units specialized in the care of patients with pDoC, which may have facilitated the accuracy of the clinical evaluation. Further studies should include less experienced rehabilitation departments and intensive care units in the validation to better measure the ability of the SECONDs to be used in all clinical settings. Also, the predictive value of the SECONDs in comparison with the CRS-R alone and combined with instrumental evaluations should be explored in further studies.

This study provided a cross-culturally adapted and validated Italian version of the SECONDs that can be applied to assess individuals with pDoC in the Italian context. The present multicentre validation study confirmed that the Italian version of the SECONDs is valid, with nearly perfect reliability, substantial interrater reliability, and substantial concurrent validity compared to the CRS-R.

## Supporting information

S1 File IT-SECONDs scale. (PDF)

S2 File IT-SECONDs instructions. (PDF)

S3 File IT-SECONDs indice aggiuntivo. (PDF)

S4 File SECONDs English protocol. (PDF)

S5 File SECONDs French protocol. (PDF)

## Abbreviations

DoC: Disorders of Consciousness
UWS: Unresponsive Wakefulness Syndrome
MCS: Minimally Conscious State
EMCS: emergence from the Minimally Conscious State
MCS: Minimally Conscious State Minus
MCS+: Minimally Conscious State Plus
pDoC: prolonged DoC
CRS-R: Coma Recovery Scale-Revised
SECONDs: Simplified Evaluation of CONsciousness Disorders
