# Supplementary material for: Cross-cultural adaptation and multicentric validation of the Italian version of the Simplified Evaluation of CONsciousness Disorders (SECONDs)
Source: PLoS One. 2025 Feb 10;20(2):e0317626. doi: 10.1371/journal.pone.0317626 (PMC11809904; doi:10.1371/journal.pone.0317626)
Supplement: S1 File — (PDF) [file pone.0317626.s001.pdf]

Paziente:__________________ Esaminatore:________________ Data:__________________ Ora: ____________________

Valutazione semplificata dei disturbi della coscienza (Simplified Evaluation of CONsciousness Disorders, SECONDs)

____
____

A. Osservazione

Comando 1: ________________________________ .../3
2: ________________________________ .../3
3: ________________________________ .../3
Comando scritto: ____________________________ .../3
➔ Il paziente risponde almeno 2 volte a uno dei comandi (= punteggio 6)

☐ **B. Risposta al comando** (punteggio 6)
3 x 3 comandi verbali
Intervallo di 10” tra i comandi
(1 x 3 comandi scritti se 0/3),
Stop se 2 comandi 3/3

Se risposta al comando

Codice sì: ________________________________
Codice no: ________________________________

Risposte: .../5 □ verbali □ autobiografiche

Corrette: .../5 □ scritte □ contestuali

➔ Il paziente risponde (anche erroneamente) ad almeno 3 domande (= punteggio 7)

➔ Il paziente risponde correttamente alle 5 domande (= punteggio 8)

C. Comunicazione
☐ **Intenzionale** (punteggio 7)

☐ **Funzionale** (punteggio 8)
Domande autobiografiche
Nome (no), data di nascita (sì), nome (sì), data di nascita (no), figli (sì/no)
Se le risposte sono sbagliate: domande contestuali *Luogo (sì), indossa il cappello (sì), luogo (no), toccargli il viso (sì), toccargli il viso (no)*

Orizzontale: .../2 Verticale: .../2

□ spontaneo □ con specchio □ Apertura manuale degli occhi
➔Il paziente effettua almeno 2 inseguimenti visivi di almeno 2 secondi (= punteggio 4)

☐ **D. Inseguimento visivo** (punteggio 4)
Persona/specchio, a 30 cm dal viso; ogni movimento sull’asse orizzontale o verticale = 4” (→ ← ↓ ↑)

Sup S: .../1 Sup D: .../1
Inf S: .../1 Inf D: .../1

□ spontanea □ con specchio □ Apertura manuale degli occhi
➔Il paziente effettua almeno 2 fissazioni dello sguardo di almeno 2 secondi (= punteggio 3)

☐ **E. Fissazione visiva** (punteggio 3)
Persona/specchio, 30 cm dal viso; stimolo presentato in ogni quadrante

Se nessuna risposta al

Posizione: S: .../1 D: .../1
Anticipazione: S: .../1 D: .../1
➔Il paziente si avvicina all’area stimolata almeno 1 volta con la mano non stimolata (= punteggio 2)
➔Il paziente presenta 2 anticipazioni (= punteggio 6)

☐ **F. Localizzazione del dolore** (punteggio 2)
Informare il paziente
5” di pressione sul letto ungueale, 1 prova su ogni mano

co ma ndo

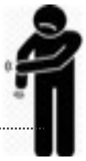

Nota bene:…… ➔Il paziente presenta almeno 1 comportamento finalizzato (= punteggio 5)

☐ **G. Comportamenti finalizzati** (punteggio 5)

Esempio: grattarsi, afferrare le lenzuola, afferrare le sponde del letto, ridere o piangere in modo contestualizzato, eccetera.

0-25%/25-50%/50-75%/75-100%<br>□ spontaneamente<br>□ stimolazione uditiva/tattile/al dolore<br>➔ Il paziente esegue almeno 1 apertura degli occhi durante l'intera valutazione (= punteggio 1)

H. Vigilanza

☐ **Apertura degli occhi** (punteggio 1)

☐ **Nessuna vigilanza** (punteggio 0)

Annotare la percentuale del tempo di apertura degli occhi e le stimolazioni effettuate

Diagnosi: Coma (0)/SV(1)/SCM- (2-5)/SCM+ (6-7)/ESCM (8)

Indice aggiuntivo: …/100
