# Supplementary material for: Cross-cultural adaptation and multicentric validation of the Italian version of the Simplified Evaluation of CONsciousness Disorders (SECONDs)
Source: PLoS One. 2025 Feb 10;20(2):e0317626. doi: 10.1371/journal.pone.0317626 (PMC11809904; doi:10.1371/journal.pone.0317626)
Supplement: S2 File — (PDF) [file pone.0317626.s002.pdf]

Valutazione semplificata dei disturbi della coscienza (Simplified Evaluation of CONsciousness Disorders, SECONDs)

Istruzioni per la valutazione (tappe da seguire nell’ordine)

Osservazione

Osservazione del paziente per un minuto: prendere nota dei comportamenti spontanei del paziente. Risvegliare il paziente (mediante stimolazioni uditive, tattili o dolorose) se non mostra un’apertura prolungata degli occhi. Durante la valutazione, cercare di far aprire gli occhi (se possibile) prima di somministrare ciascun item. Se non è possibile, somministrare gli item visivi (inseguimento e fissazione) e la capacità di guardare in alto a comando, sollevando manualmente le palpebre del paziente.

Attenzione! Durante tutta la valutazione osservare il paziente:

- **Vigilanza:**
  - Apre gli occhi? Spontaneamente o dopo stimolazione? In che percentuale di tempo il paziente ha tenuto gli occhi aperti?
  - Annotare il numero di ciascun tipo di stimolazione applicata.
- **Comportamenti finalizzati:**
  - Presenta comportamenti finalizzati a se stesso o al suo ambiente, non riflessi e non conseguenti a un comando? Questi comprendono, ma non sono limitati a: il paziente si gratta, afferra le coperte, afferra le sponde del letto, si tira i vestiti, tira la cannula tracheostomica/gastrostomica/il catetere vescicale, avvicina la mano alla bocca per tossire/sbadigliare, sorride/ride/piange in modo adeguato al contesto, risponde verbalmente o a gesti quando gli si parla (non necessariamente in modo corretto) o qualsiasi altro comportamento automatico. Registrare il numero di volte in cui uno o più di questi comportamenti vengono osservati.

Risposta al comando

Come somministrarlo?

L’esaminatore chiede tre volte al paziente di eseguire un movimento particolare (= un comando) che non esegue spontaneamente in maniera ripetitiva (cfr. periodo di osservazione). Devono essere valutati almeno tre diversi comandi (richiesti tre volte ciascuno, lasciando un minimo di 10 secondi tra i comandi). Questo può essere ripetuto immediatamente per ottimizzare la motivazione del paziente. L’esaminatore può chiedere di:

- Muovere un arto, un’estremità o la testa
- Chiudere gli occhi con forza e per un tempo prolungato
- Sbattere le palpebre due volte

- Guardare verso un punto specifico/guardare in alto
- Aprire o chiudere la bocca
- Dire una parola/produrre un suono
- Fare sì/no con la testa
- Tirar fuori la lingua
- Stringere la mano
- Premere un campanello o equivalente

Un'eccezione alla regola dei tre comandi può essere fatta qualora i primi due comandi siano eseguiti senza errori da parte del paziente (3/3 richieste).

In caso di sordità nota o sospetta, i comandi possono essere somministrati per iscritto. Allo stesso modo, se il paziente non reagisce ai comandi vocali, è imperativo somministrare almeno un comando scritto, dopo essersi assicurati dell'integrità della via visiva.

Come attribuire il punteggio?

Assegnare *"6" punti (risposta al comando)* se il paziente risponde correttamente ad almeno due richieste per lo stesso comando. È necessario che queste risposte siano inequivocabile e siano state prodotte perché richieste, e non spontaneamente. Pertanto, qualsiasi movimento di ridotta ampiezza, ambiguo o dovuto a uno spasmo o a un riflesso di prensione, non sarà conteggiato. Riportare sul modulo i comandi utilizzati e il numero di volte che il paziente ha reagito correttamente.

Comunicazione (item sottomesso a condizione)

Quando e come valutarlo?

Eseguirlo se il paziente può esprimere un "sì" e un "no" verbalmente, spontaneamente o meno, con un gesto o per iscritto, oppure se due diverse risposte al comando hanno esito positivo. In quest'ultimo caso è necessario stabilire un codice basato sulle risposte al comando osservate precedentemente. È importante spiegare chiaramente al paziente che un primo movimento significa "sì" e un secondo movimento significa "no". Un'assenza di movimento non può essere considerata un sì o un no, sono necessari due movimenti ben distinti. Verificare che il paziente possa esprimere un "sì" e un "no". Quando il codice è ben chiaro, lo sperimentatore pone al paziente le seguenti 5 domande autobiografiche semplici e chiuse. L'esaminatore può ricordare il codice del "sì" e "no" prima di ogni domanda.

- Il suo nome è "*(nome falso)*"?
- Lei è nato nel "*(anno di nascita corretto)*"?
- Il suo nome è "*(nome corretto)*"?
- Lei è nato nel *"(anno errato)*"?
- Ha figli?

Se il paziente sembra avere difficoltà con queste domande, porre le seguenti domande contestuali semplici:

- Siamo in "*(luogo della valutazione: ospedale, casa di cura, ecc.)*"?
- Ho un cappello in testa?
- Siamo in piscina?
- Le sto toccando il viso? (toccargli il viso)
- Le sto toccando il viso? (non toccargli il viso)

Come attribuire il punteggio?

Assegnare *"7" punti (comunicazione intenzionale)* se il paziente ha risposto ad almeno 3 domande sulle 5 poste, importa poco se la risposta sia stata corretta o meno. Assegnare *"8" punti (comunicazione funzionale)* se il paziente ha risposto correttamente alle 5 domande (indipendentemente se dalla serie autobiografica o contestuale). Riportare sul modulo il codice e il numero di risposte corrette/errate. Annotare le domande poste.

Inseguimento visivo

Come valutarlo?

Il paziente segue spontaneamente e chiaramente con lo sguardo l'esaminatore che si sposta silenziosamente intorno al letto, per almeno due secondi e due volte.

Se non si osserva spontaneamente un inseguimento visivo chiaro, utilizzare uno specchio. *Prima di mostrare lo specchio, lo sperimentatore deve avvertire il paziente che sta per vedere sé stesso, e che sul suo viso o nell'ambiente circostante potrebbero esserci elementi inaspettati (sonde, bende, ferite, ecc.).* Posizionare uno specchio davanti al paziente (in linea con il suo sguardo) per catturarne l'attenzione, a circa 30 cm dal suo viso. Spostare lo specchio lentamente (assicurandosi che il paziente possa vedersi riflesso in ogni momento), da sinistra a destra (o viceversa, a seconda della direzione iniziale del suo sguardo), da destra a sinistra, dall'alto verso il basso, quindi dal basso verso l'alto. Ogni movimento (ad es. da sinistra a destra) deve durare almeno 4 secondi.

Come attribuire il punteggio?

Assegnare *"4" punti (inseguimento visivo)* se si osserva un inseguimento continuo per almeno due secondi e due volte. Riportare sul modulo il numero di inseguimenti (minimo due secondi) osservati su ciascun asse.

Fissazione visiva

Come valutarlo?

Il paziente fissa chiaramente l'esaminatore silenzioso o qualsiasi altro oggetto che appare nel suo campo visivo, per due secondi e due volte. È importante che l'oggetto della fissazione (o l'esaminatore) non sia inizialmente e spontaneamente nell'asse del suo sguardo.

Se non si osserva una fissazione chiara e spontanea, utilizzare uno specchio. *Prima di mostrare lo specchio, lo sperimentatore deve avvertire il paziente che sta per vedere sé stesso, e che sul suo viso o nell'ambiente circostante potrebbero esserci elementi inaspettati (sonde, bende, ferite, ecc.).* Mostrare uno specchio nel campo visivo del paziente, in luoghi diversi, ma non nell'asse del suo sguardo (testare i 4 quadranti del campo visivo). Lo specchio deve trovarsi a circa 30 cm dal suo viso. Assicurarsi che lo specchio sia rivolto verso il viso del paziente.

Come attribuire il punteggio?

Assegnare *"3" punti (fissazione visiva)* se si osservano due fissazioni della durata minima di due secondi. È necessario osservare un netto cambiamento di orientamento dello sguardo verso l'oggetto/persona, seguito da una fissazione di almeno due secondi. Riportare sul modulo i quadranti in cui il paziente ha fissato lo sguardo.

Localizzazione del dolore (item sottomesso a condizione)

Questo item deve essere somministrato SOLO se il paziente non ha ottenuto alcun punteggio maggiore di "5".

Come valutarlo?

a. L'esaminatore posiziona una penna o una matita perpendicolarmente al letto ungueale della mano del paziente e attende cinque secondi senza dire nulla.
b. Avverte il paziente che gli farà male, ma che può evitarlo ritirando la mano.
c. Se il paziente non toglie la mano entro cinque secondi, l'esaminatore esercita una pressione significativa con la penna o la matita sul letto ungueale del paziente per cinque secondi. Eseguire una prova su ciascuna mano.

Come attribuire il punteggio?

Assegnare *"2" punti (localizzazione del dolore)* se durante almeno una delle prove, l'altra mano del paziente va a toccare chiaramente la mano stimolata. Se invece il paziente ha chiaramente ritirato la mano DOPO la minaccia (b), e non durante i 5 secondi precedenti (a), in entrambi i test, assegnare *"6" punti (risposta al comando).*

Vigilanza

Come attribuire il punteggio?

Al termine della valutazione, assegnare *"0" punti (non vigile)* se per tutto l'esame il paziente non apre mai gli occhi, indipendentemente dalla stimolazione (compreso il dolore).

Assegnare *"1" punto (Vigile)* se il paziente apre gli occhi almeno una volta durante la valutazione. Specificare se l'apertura degli occhi avviene:

A. Al dolore
B. Alla stimolazione tattile
C. Alla stimolazione uditiva
D. Spontaneamente

Specificare il numero di stimolazioni di ciascun tipo e in che percentuale di tempo gli occhi del paziente erano aperti durante la valutazione: 0-25%; 25-50%; 50-75%; 75-100%.

Comportamenti finalizzati

Come attribuire il punteggio?

Assegnare *"5" punti (comportamenti finalizzati)* se il paziente mostra almeno un comportamento finalizzato, qualunque esso sia. Riportare sul modulo i comportamenti osservati nonché il numero di osservazioni per ciascuno di essi. A titolo di promemoria, all'inizio di questo manuale vengono forniti degli esempi.

SECONDs: sintesi

1. **Osservazione** (un minuto): annotare i comportamenti spontanei del paziente.

   Durante tutta la valutazione: osservare l'apertura degli occhi del paziente e se mostra comportamenti finalizzati.

2. **Risposta al comando**: scegliere almeno tre comandi (movimenti che il paziente non esegue spontaneamente), tre tentativi per ogni comando. Assegnare "6" punti se viene effettuato almeno un comando per almeno due volte su tre richieste.
3. **Comunicazione** (*in caso di codice per risposta al comando o comunicazione spontanea vocale o gestuale*): cinque semplici domande autobiografiche di cui l'esaminatore conosce la risposta, assegnare "7" punti se vengono fornite almeno tre risposte (anche se errate), "8" punti in caso di cinque risposte corrette. Se le domande autobiografiche risultano inconcludenti, provare le domande contestuali.
4. **Inseguimento visivo**: in assenza di inseguimento spontaneo dell'esaminatore, attirare lo sguardo del paziente con uno specchio. Spostarlo da sinistra a destra poi da destra a sinistra (o viceversa se il paziente guarda a destra); dall'alto verso il basso e poi dal basso verso l'alto. Ogni movimento (da un estremo all'altro) deve durare quattro secondi; assegnare "4" punti se si osservano almeno due episodi di inseguimento di due secondi.
5. **Fissazione visiva**: in assenza di fissazione spontanea dell'esaminatore, mostrare uno specchio in ogni quadrante del campo visivo del paziente. Assegnare "3" punti se il paziente cambia la direzione dello sguardo per fissare lo specchio per almeno due secondi.
6. *Se il paziente non ha raggiunto un punteggio maggiore di "5"*, valutare la **localizzazione del dolore**: premere il letto ungueale della mano del paziente (una volta per mano), avvertendolo che gli farà male. Assegnare "2" punti se il paziente lo localizza con l'altra mano almeno una volta. Assegnare "6" punti se il paziente anticipa chiaramente, ritirando la mano dopo aver sentito la minaccia, per due volte.
7. Al termine della valutazione: assegnare i punti per la **vigilanza** e i **comportamenti finalizzati.**
   a. Vigilanza: assegnare "1" punto se ha aperto gli occhi almeno una volta, altrimenti assegnare "0" punti. Specificare quale stimolazione è stata necessaria e la percentuale di tempo in cui ha tenuto gli occhi aperti.
   b. Comportamenti finalizzati: assegnare "5" punti se il paziente presenta almeno un episodio di comportamenti finalizzati inequivocabile.
