# Supplementary material for: Cross-cultural adaptation and multicentric validation of the Italian version of the Simplified Evaluation of CONsciousness Disorders (SECONDs)
Source: PLoS One. 2025 Feb 10;20(2):e0317626. doi: 10.1371/journal.pone.0317626 (PMC11809904; doi:10.1371/journal.pone.0317626)
Supplement: S3 File — (PDF) [file pone.0317626.s003.pdf]

Valutazione semplificata dei disturbi della coscienza (Simplified Evaluation of CONsciousness Disorders, SECONDs)

Paziente: ________________			Data: ___/ ____/ ______ dalle ___:___ alle ___:___		Effettuata da: __________________		
Diagnosi	Livello	Item	Specificità	N.	Max	OK?	Criteri
COMA	0	Nessuna vigilanza		-	-		
UWS/VS	1	Vigilanza	Al dolore	-	-		
			Alla stimolazione tattile	-	-		
			Alla stimolazione uditiva	-	-		
			Spontaneamente	-	-		
MCS-	2	Localizzazione del dolore	Mano destra		1		min. 1 localizz.
			Mano sinistra		1		
MCS-	3	Fissazione visiva	In alto a sinistra		1		min. 2 fissazioni (min 2 sec)
			In alto a destra		1		
			In basso a sinistra		1		
			In basso a destra		1		
MCS-	4	Inseguimento visivo	Asse orizzontale		2		min. 2 insegui-menti (min 2 sec)
			Asse verticale		2		
MCS-	5	Comportamenti finalizzati	Comportamento: ________________________		-		min. 2 oss. (qualsiasi movi-mento)
			Comportamento: ________________________		-		
			Comportamento: ________________________		-		
			Comando 1: __________________________		3		

Valutazione semplificata dei disturbi della coscienza (Simplified Evaluation of CONsciousness Disorders, SECONDs)

MCS+	6	Risposta al comando	Comando 2: ____________________________		3		min. 2/3 riusciti per uno stesso ordine
			Comando 3: ____________________________		3		
			Comando 4: ____________________________		3		
MCS+	7	Comunicazione intenzionale	Specificare il codice: ___________________	-	-		Da 3 a 5 risposte, anche se errate
			Risposte errate		5		
			Risposte corrette		4		
EMCS	8	Comunicazione funzionale	Risposte corrette		5		5 corrette

Osservazioni e note:	PUNTEGGIO TOTALE: ______<br>DIAGNOSI: _________________<br>Indice aggiuntivo: ______

Come calcolare l'indice aggiuntivo?

Per ogni livello, indicare il punteggio ponderato secondo la seguente tabella di corrispondenza:

Valutazione semplificata dei disturbi della coscienza (Simplified Evaluation of CONSciousness Disorders, SECONDs)

Livello	Punteggio ponderato
0	0
1	___
2	___
3	___
4	___
5	___
6	___
7 o 8	___

Sommare i punteggi ponderati.
L’indice massimo è 100.

1	Vigilanza	Al dolore	1
		Alla stimolazione tattile	2
		Alla stimolazione uditiva	3
		Spontaneamente	4
2	Localizzazione del dolore	Su una mano	2
		Su entrambe le mani	4
3	Fissazione visiva	In due occasioni	6
		In tre occasioni	9
		In quattro occasioni (tutte)	12
4	Inseguimento visivo	In due occasioni	8
		In tre occasioni	12
		In quattro occasioni (tutte)	16
5	Comportamenti finalizzati	Un movimento	5
		Due movimenti diversi	10
		Più di due movimenti diversi	15
6	Risposta al comando	1 comando 2/3	6
		1 comando 3/3	12
		2 comandi 2/3	18
		2 comandi 3/3	24
7	Comunicazione intenzionale	3 o 4 risposte (anche errate)	7
		5 risposte (anche errate)	14
		3 o 4 risposte (corrette)	21
8	Comunicazione funzionale	5 risposte (corrette)	29
