# Supplementary material for: Cross-cultural adaptation and multicentric validation of the Italian version of the Simplified Evaluation of CONsciousness Disorders (SECONDs)
Source: PLoS One. 2025 Feb 10;20(2):e0317626. doi: 10.1371/journal.pone.0317626 (PMC11809904; doi:10.1371/journal.pone.0317626)
Supplement: S4 File — (PDF) [file pone.0317626.s004.pdf]

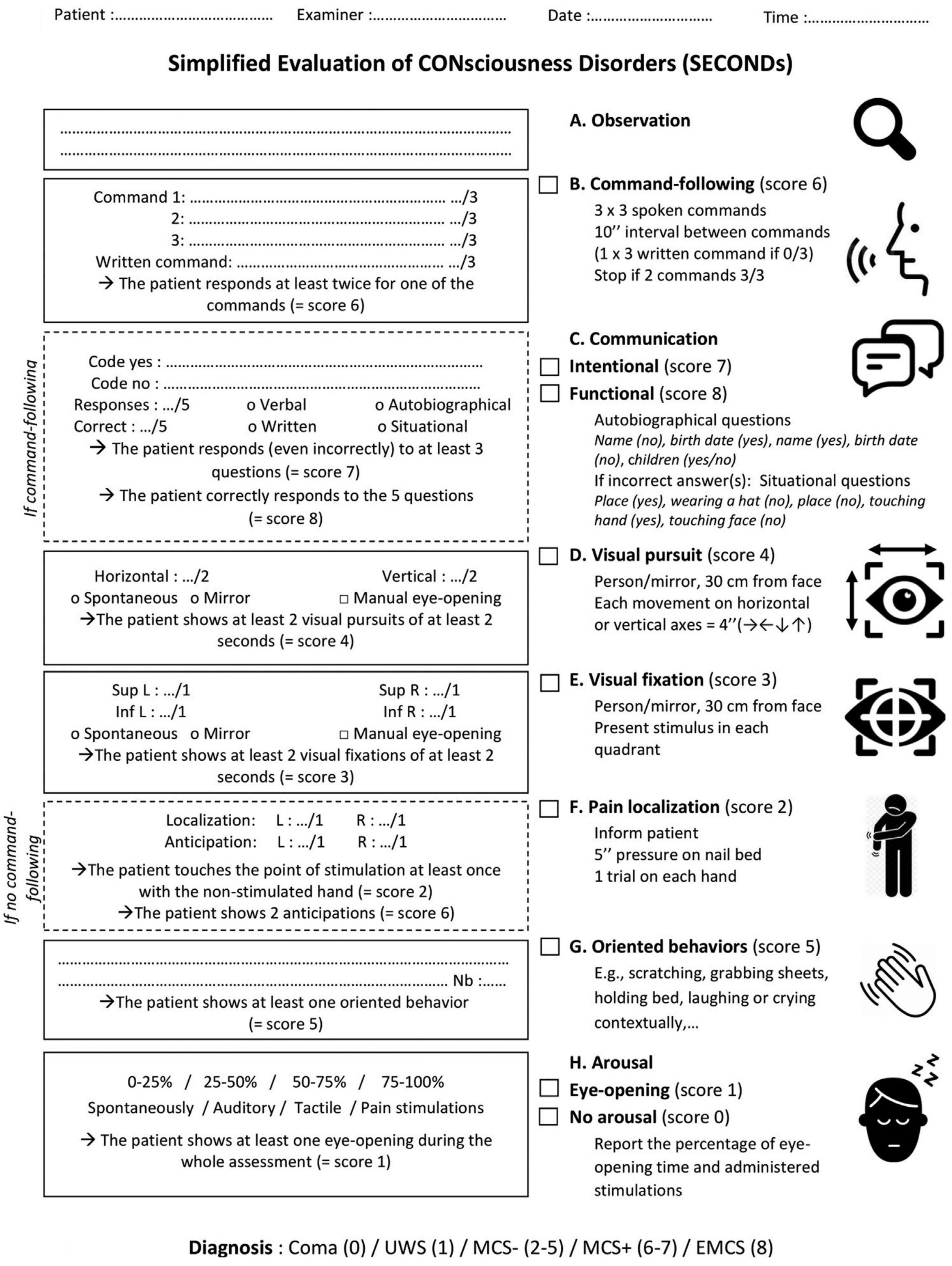

Fig. 1. Administration of the Simplified Evaluation of CONsciousness Disorders (SECONDs). We recommend administration of at least 5 SECONDs in a short time period (e.g., 10 days) to reduce misdiagnosis rates [11].

R/SECONDs and SECONDs/SECONDs) was randomized, so that half of the participants were assessed with the CRS-R on the first day and the other half on the second day. Three examiners were randomly assigned to the 4 evaluations (Fig. 2). The administration duration of each assessment was recorded.

The examiners were blinded to the participants' medical diagnosis and medical history and remained blinded regarding the results and type of commands previously tested during all other behavioral assessments, including both SECONDs and CRS-R.

2.4. Statistical analyses

Descriptive statistics were used for participant characteristics. Age and time since injury are expressed as mean (SD) and sex and
