# Supplementary material for: Cross-cultural adaptation and multicentric validation of the Italian version of the Simplified Evaluation of CONsciousness Disorders (SECONDs)
Source: PLoS One. 2025 Feb 10;20(2):e0317626. doi: 10.1371/journal.pone.0317626 (PMC11809904; doi:10.1371/journal.pone.0317626)
Supplement: S5 File — (PDF) [file pone.0317626.s005.pdf]

Patient(e) :…………………………… Evaluateur :…………………………… Date :………………………… Heure :…………………………

Simplified Evaluation of CONsciousness Disorders (SECONDs)

A. Observation

………………………………………………………………………………………………
………………………………………………………………………………………………

☐ **B. Réponse à la commande** (score 6)

3 x 3 commandes verbales
10'' intervalle entre commandes
(1 x 3 commandes écrites si 0/3)
Stop si 2 commandes 3/3

Commande 1: ……………………………………………………… …/3
2: ……………………………………………………… …/3
3: ……………………………………………………… …/3
Commande écrite: ………………………………………………… …/3
→ Le patient répond au moins 2x à l'une des commandes (= score 6)

Si réponse à la commande

C. Communication

☐ **Intentionnelle** (score 7)

☐ **Fonctionnelle** (score 8)

Questions autobiographiques
Prénom (non), date naissance (oui), prénom (oui), date naissance (non), enfants (oui/non)
Si réponses erronées: questions situationnelles
Lieu (oui), porter chapeau (oui), lieu (non), toucher main (oui), toucher visage (non)

Code oui : ……………………………………………………………………
Code non : ……………………………………………………………………

Réponses : …/5 o Verbales o Autobiographiques
Correctes : …/5 o Ecrites o Situationnelles

→ Le patient répond (même erronément) à au moins 3 questions (= score 7)
→ Le patient répond correctement aux 5 questions (= score 8)

☐ **D. Poursuite visuelle** (score 4)

Personne/miroir, 30 cm du visage
Chaque mouvement sur l'axe horizontal ou vertical = 4''
(→←↓↑)

Horizontal : …/2 Vertical : …/2
o Spontanée o Miroir □ Ouverture manuelle des yeux
→Le patient présente au moins 2 poursuites visuelles d'au moins 2 secondes (= score 4)

☐ **E. Fixation visuelle** (score 3)

Personne/miroir, 30 cm du visage
Stimulus présenté dans chaque quadrant

Sup G : …/1 Sup D : …/1
Inf G : …/1 Inf D : …/1
o Spontanée o Miroir □ Ouverture manuelle des yeux
→Le patient présente au moins 2 fixations visuelles d'au moins 2 secondes (= score 3)

Si aucune réponse à la commande

☐ **F. Localisation à la douleur** (score 2)

Informer le patient
5'' pression sur lit de l'ongle
1 essai sur chaque main

Localisation: G : …/1 D : …/1
Anticipation: G : …/1 D : …/1
→Le patient approche la zone stimulée au moins 1x avec la main non stimulée (= score 2)
→Le patient présente 2 anticipations (= score 6)

☐ **G. Comportements orientés** (score 5)

Ex: se gratter, saisir les draps, tenir le lit, rire ou pleurer de manière contextualisée,…

………………………………………………………………………………………………
……………………………………………………………………………… Nb :……
→Le patient présente au moins 1 comportement orienté (= score 5)

H. Eveil

☐ **Ouverture des yeux** (score 1)

☐ **Aucun éveil** (score 0)

Noter le pourcentage de temps d'ouverture des yeux et les stimulations administrées

0-25% / 25-50% / 50-75% / 75-100%
o Spontanément
o Stimulation Auditive / Tactile / Douleur
→ Le patient présente au moins 1 ouverture des yeux durant l'ensemble de l'évaluation (= score 1)

Diagnostic : Coma (0) / ENR (1) / ECM- (2-5) / ECM+ (6-7) / EECM (8)
Index additionnel : … /100
